# CD4 T cells correlate with better prognosis in medulloblastoma

**DOI:** 10.3389/fonc.2025.1593329

**Published:** 2025-06-12

**Authors:** Jin Zhang, Siqi Ren, Shuting Li, Yuan Wang, Lulu Wan, Wenchao Gao, Huaying Sun, Xiaojun Gong, Miao Li, Yanling Sun, Liming Sun, Zhigang Li, Tianyou Wang, Shuxu Du, Wanshui Wu

**Affiliations:** ^1^ Department of Pediatrics, Beijing Shijitan Hospital, Capital Medical University, Beijing, China; ^2^ Hematologic Disease Laboratory, Hematology Center, Beijing Key Laboratory of Pediatric Hematology Oncology, National Key Discipline of Pediatrics (Capital Medical University), Key Laboratory of Major Disease in Children, Ministry of Education, Beijing Pediatric Research Institute, Beijing Children’s Hospital, Capital Medical University, National Center for Children’s Health, Beijing, China; ^3^ Hematology Center, Beijing Key Laboratory of Pediatric Hematology Oncology, National Key Discipline of Pediatrics (Capital Medical University), Key Laboratory of Major Disease in Children, Ministry of Education, Beijing Children’s Hospital, Capital Medical University, National Center for Children’s Health, Beijing, China

**Keywords:** medulloblastoma, CD4 T cells, CD8 T cells, programmed death 1, programmed death-ligand 1, prognosis

## Abstract

**Objective:**

T cells and tumor-associated macrophages (TAMs) are critical immune components within the brain tumor microenvironment (TME), yet their precise roles in medulloblastoma remains unclear. In this study, we examined the infiltration characteristics of T cells in medulloblastoma tissues and analyzed the correlation between T cells and the clinical outcomes of medulloblastoma patients. Additionally, we further investigated the relationship between T cells and TAMs.

**Methods:**

We enrolled a total of 72 patients diagnosed with medulloblastoma and subsequently detected the T cell makers and programmed death 1/programmed death-ligand 1 (PD-1/PD-L1) in paraffin-embedded sections using multiple immunofluorescence staining method. The correlation between T cell infiltration, clinical characteristics and prognosis were analyzed. Finally, we used Spearman correlation analysis to evaluate the correlation between T cells and TAMs.

**Results:**

The median age at diagnosis of 72 patients (54 boys, 18 girls) was 7.5 years (range: 0.8–18 years). These patients included 43 cases of classic medulloblastoma (CMB), 24 cases of desmoplastic/nodular medulloblastoma (DNMB), 2 cases of medulloblastoma with extensive nodularity (MBEN) and 3 cases of large-cell/anaplastic medulloblastoma (LCA). The molecular subgroups consisted of 3 wingless (WNT), 29 sonic hedgehog (SHH) and 40 non-WNT/non-SHH cases. Twenty-five cases presented with metastasis at diagnosis, while 47 cases were without metastasis. Thirteen cases exhibited with high-risk genetic abnormalities. The total T cells (*P* = 0.031) and CD4 T cells (*P* = 0.045) were significantly elevated in the SHH subgroup compared to those in the non-WNT/non-SHH subgroup. Patients with increased CD4 T cells had better 5-year PFS (*P* = 0.000) and OS (*P* = 0.001), while patients without metastasis showed better 5-year PFS (*P* = 0.031) and OS (*P* = 0.015). Multivariate analysis showed that CD4 T cells were an independent prognostic factor affecting both the 5-year PFS (*P* = 0.004, HR = 0.230, 95% CI = 0.085-0.662) and OS (*P* = 0.017, HR = 0.180, 95% CI = 0.044-0.739). Additionally, it was observed that CD4 T cells exhibited a positive correlation with M_total_ (total macrophages) (*P* < 0.05, r = 0.249) and M_mix_ (M1/M2 mixed phenotype macrophages) (*P* < 0.01, r = 0.325), and CD3^+^CD8^+^PD-1^+^ cells showed a positive correlation with M_mix_ (*P* < 0.05, r = 0.258).

**Conclusion:**

The increase in CD4 T cells predicts a better prognosis in medulloblastoma patients, particularly within the SHH and non-WNT/non-SHH subgroups, and they may serve as a potential therapeutic target for medulloblastoma. Additionally, there may be a potential interaction between CD4 T cells and TAMs that warrants further investigation.

## Introduction

Immune cells represent a critical component of the brain tumor microenvironment (TME). Elucidating their functions and interactions holds substantial significance for understanding tumor initiation, progression and metastasis. Pediatric medulloblastomas are the most commonly diagnosed embryonal tumors of the central nervous system (1). Within the immune microenvironment of medulloblastoma, T cells and macrophages are the predominant immune cell types (2). Collectively, these cell populations constitute essential elements of the TME and exhibit complex characteristics, capable of either inducing inflammatory responses or exerting suppressive effects (3, 4). Based on our investigation of macrophages, we conducted an in-depth analysis of the infiltration characteristics of T cells and examined the relationship between these two cell types.

T cells are important immune cells in the TME and can be classified into CD4 T cells and CD8 T cells. CD4 T cells, also known as helper T cells (Th), play a significant role in cellular immunity. They can assist CD8 T cells in executing cellular immune responses to eliminate tumor cells. Additionally, CD4 T cells can provide essential support for humoral immunity and exert direct cytotoxic effects on tumor cells (5). Regulatory T cells (Tregs), a subset of CD4 T cells characterized by the phenotype CD4^+^FoxP3^+^ (6), play an important role in modulating immune responses. CD8 T cells, also known as cytotoxic T lymphocytes (CTLs), exhibit cytotoxic activity against tumor cells. Programmed death 1 (PD-1) and its ligand, programmed death-ligand 1 (PD-L1), transmit inhibitory signals to T cells, serving as critical immune checkpoints that suppress the activity of CD8 T cells (7). Both T cells and macrophages are recruited to the TME to interact. Research has demonstrated that tumor-associated macrophages (TAMs) can inhibit the function of CD8 T cells (8).

In medulloblastoma, it is reported that the total T cells are comparatively higher in sonic hedgehog (SHH) subgroup (2, 9). Furthermore, researches have demonstrated that the expression of PD-1/PD-L1 is generally inactive in pediatric tumors (10, 11). However, the relationship between tumor-infiltrating T cells and the prognosis of medulloblastoma patients remains inconclusive. A study has shown that there is no significant correlation between T cells and overall survival in medulloblastoma patients (12), whereas another research has suggested that a reduction in CD8 T cells may be indicative of a poorer prognosis for these patients (13). Therefore, further investigation is warranted to elucidate the role of T cells in medulloblastoma.

In this study, we conducted a comprehensive analysis to investigate the correlation between T cells and both the clinical characteristics and outcomes in pediatric patients with medulloblastoma. Additionally, we further explored the relationship between T cells and TAMs on the basis of our previous research (14).

## Materials and methods

### Patients and samples

A total of 72 pediatric patients diagnosed with medulloblastoma were involved in this study. All patients were newly diagnosed between 2015 and 2020. At the time of surgery, all patients were treatment-naive and subsequently underwent chemotherapy and/or craniospinal irradiation following the surgical procedure. Treatment was performed according to the German Society of Pediatric Oncology and Hematology (GPOH) Protocol HIT-2000 (15, 16). Patient information, including gender, age of onset, pathological subtype, molecular subtype, tumor stage and treatment outcomes were collected via a comprehensive review of medical records conducted by researchers who were independent of the experimental procedures. This study was approved by the Ethics Committee of Beijing Shijitan Hospital, and informed consent was obtained from all of the patients and/or their parents.

### Multiple immunofluorescence staining

Paraffin-embedded tissue sections with a thickness of 5 µm were prepared and subsequently stained for CD3, CD8, CD4, FoxP3 and PD-1/PD-L1 using a multiplex immunofluorescence technique. Multiplex immunofluorescence staining was conducted using PANO 4-plex IHC kit (catalog number: 0001100020, Panovue, Beijing, China), comprising monochromatic fluorescent dyes (Opal 520, Opal 570, Opal 650 and DAPI), a signal amplification reaction buffer, and a secondary antibody (polymer-conjugated horseradish peroxidase (HRP) anti-mouse/rabbit IgG). The primary antibodies utilized in this study were rabbit anti-human CD3 (catalog number: 0088100050), rabbit anti-human CD8 (catalog number: 0032300020), rabbit anti-human CD4 (catalog number: 0118800020), mouse anti-human FoxP3 (catalog number: 0094050050), mouse anti-human PD1 (catalog number: 0040100050), and rabbit anti-human PD-L1 (catalog number: 0109500020), all of which were sourced from Panovue, Beijing, China. Multiplex antibody panels applied in this study were as follows: panel 1:CD3 (1:100) with Opal 520 (1:100), CD8 (1:300) with Opal 570 (1:100), and PD-1 (1:100) with Opal 650 (1:100); panel 2: CD4 (1:800) with Opal 520 (1:100), FoxP3 (1:50) with Opal 570 (1:100), and PD-L1 (1:500) with Opal 650 (1:100).

The detailed experimental procedures were as follows. First, the slides were placed in a thermostatic oven set at 56 °C for 2 hours. Subsequently, the slides were deparaffinized in xylene and dehydrated in a graded ethanol series. Antigen retrieval was conducted using microwave heating in an alkaline antigen retrieval solution (catalog number: 0019020500). The slides were incubated with primary antibodies at room temperature for 1 hour, and then incubated with the secondary antibody for 10 minutes. Following this, each antigen was labeled with distinct fluorophores as above, and the immunofluorescence signal was amplified by tyramide signal amplification (TSA). Microwave antigen retrieval was performed again following each staining cycle. Finally, the nuclei were stained with DAPI (1:100) and the slides were mounted using anti-fade mountant.

### Digital image analysis

The stained slides were scanned using the Vectra-Polaris Automated Quantitative Pathology Imaging System (Akoya Biosciences, USA). First, full-scan images were captured, and then five regions (image size: 930µm×700µm) were randomly selected in the hot spot areas for detailed analysis. The proportion of fluorescent signal-positive cells was calculated using the inForm image analysis software (version 2.4.0). Subsequently, the average proportions in the five regions were determined. CD3^+^ cells represented the total T cells, CD3^+^CD8^+^ cells represented CD8 T cells, CD4^+^ cells represented CD4 T cells, and CD4^+^FoxP3^+^ cells represented Tregs.

### Statistical analysis

Statistical analysis was conducted using SPSS 24.0 with a P value < 0.05 indicating statistical significance. Variables that did not obey a normal distribution were described using the median (interquartile range, IQR), and comparisons between different groups were conducted using the Mann-Whitney U test. The Kaplan-Meier method was employed to estimate the survival rates and conduct univariate survival analyses, while the Log-rank test was utilized to compare differences in survival rates between distinct groups. Multivariate survival analyses were performed using the Cox regression model, and the hazard ratios (HR) along with their 95% confidence intervals (CI) were estimated. Spearman correlation analysis was applied to evaluate the correlation between T cells and TAMs. Progression-free survival (PFS) was defined as the interval from the date of surgery to the date of disease progression. Overall survival (OS) was defined as the interval from the date of surgery to the date of death or the last follow-up. The last follow-up time was December 31, 2024.

## Results

### Patient characteristics

A total of 72 children (54 boys, 18 girls) were enrolled in this study, and the clinical characteristics of all patients are shown in **Table 1**. The median age at diagnosis was 7.5 years (range: 0.8–18 years). Twenty cases were under 5 years old, and 52 cases aged 5 or older. These patients included 43 cases of classic medulloblastoma (CMB), 24 cases of desmoplastic/nodular medulloblastoma (DNMB), 2 cases of medulloblastoma with extensive nodularity (MBEN) and 3 cases of large-cell/anaplastic medulloblastoma (LCA). The molecular subgroups consisted of 3 wingless (WNT), 29 SHH and 40 non-WNT/non-SHH cases. There were 25 cases with metastasis and 47 cases without metastasis at the time of diagnosis, respectively. Thirteen cases exhibited with high-risk genetic abnormalities, including 6 cases with TP53 mutation and 7 cases with MYC amplification. The median follow-up time was 57.5 months (9–96 months). The 5-year PFS and OS were 55.5% ± 6.8% and 71.9% ± 5.9%, respectively.

**Table 1:** Relationship of clinical characteristics and T cells in medulloblastoma.

Characteristics	Number (%)	*P(*CD3)	*P(*CD8)	*P(*CD4)	*P(*Tregs)	*P(*PD-1)	*P(*PD-L1)
Age (year)		0.359	0.258	0.352	0.574	0.694	0.352
<5	20 (28)						
≥5	52 (72)						
Gender		0.317	0.830	0.154	0.948	0.358	0.301
boy	54 (75)						
girl	18 (25)						
Pathological type		^1^ 0.631	0.671	0.428	0.360	0.464	0.826
CMB	43 (60)	^2^ 0.459	0.542	0.177	0.073	0.455	0.302
DNMB	24 (33)	^3^ 0.683	0.766	0.181	0.117	0.654	0.297
MBEN	2 (3)						
LCA	3 (4)						
Molecular subtype		^4^ 0.031	0.121	0.045	0.094	0.198	0.813
WNT	3 (4)	^5^ 0.412	0.693	0.485	0.604	0.483	0.344
SHH	29 (40)	^6^ 0.193	0.249	0.168	0.980	0.141	0.478
non-WNT/non-SHH	40 (56)						
Metastasis		0.456	0.736	0.112	0.351	0.591	0.398
yes	25 (35)						
no	47 (65)						
High-risk gene mutation		0.994	0.924	0.619	0.557	0.363	1.000
yes	13 (18)						
no	59 (82)						

CMB, classic medulloblastoma; DNMB, desmoplastic/nodular medulloblastoma; MBEN, medulloblastoma with extensive nodularity; LCA, large-cell/anaplastic medulloblastoma. ^1^ Comparison of T cells between CMB and DNMB/MBEN; ^2^ Comparison of T cells between CMB and LCA; ^3^ Comparison of T cells between DNMB/MBEN and LCA; ^4^ Comparison of T cells between SHH and non-WNT/non-SHH; ^5^ Comparison of T cells between WNT and SHH; ^6^ Comparison of T cells between WNT and non-WNT/non-SHH.

### Association between T cells and patient characteristics

**Figure 1:**
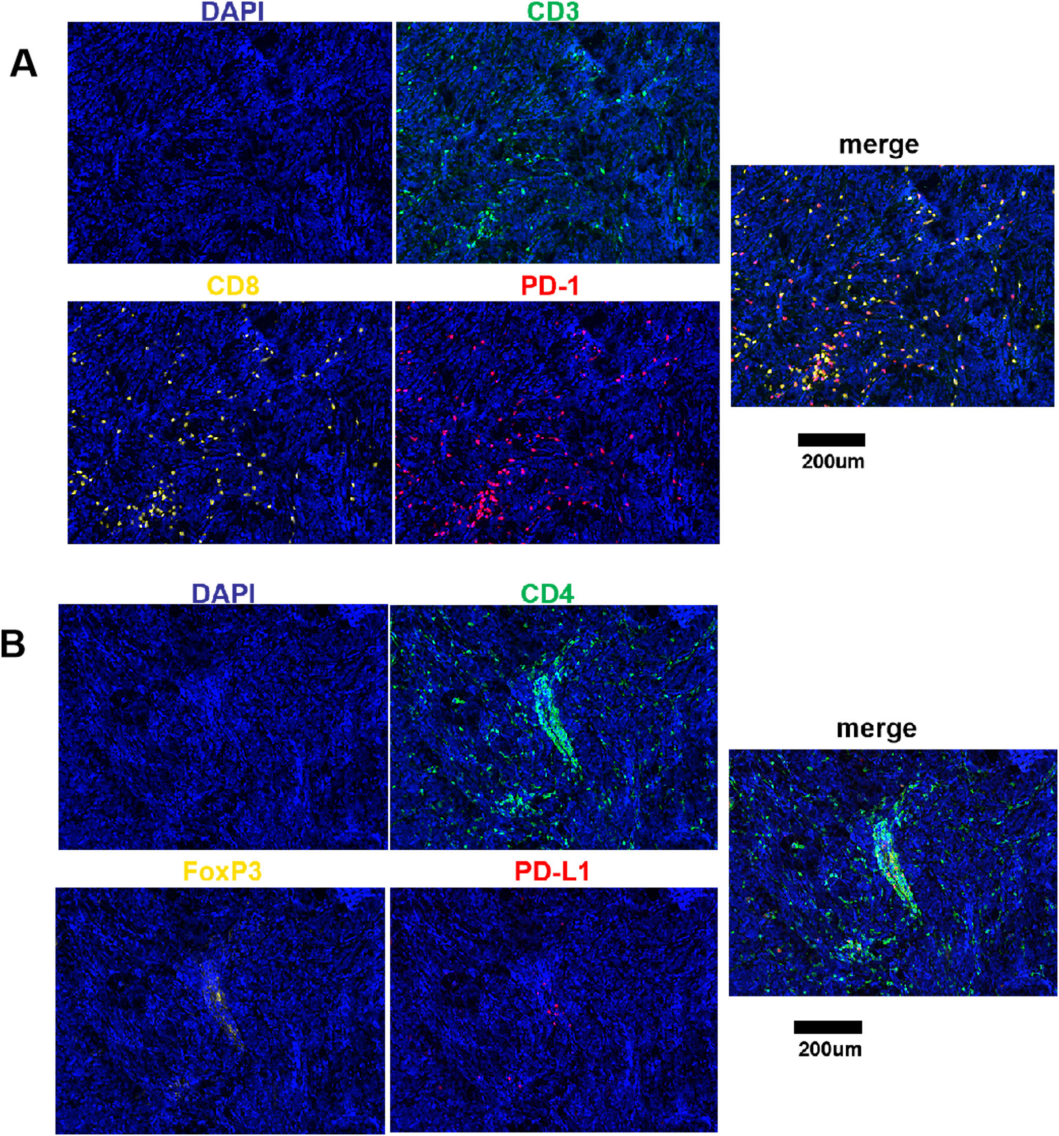
Fluorescence signal splitting and merging images. **(A)** Green staining is indicative of CD3-positive cells. Yellow staining is indicative of CD8-positive cells. Red staining is indicative of PD-1-positive cells. Blue staining is indicative of all cells. **(B)** Green staining is indicative of CD4-positive cells. Yellow staining is indicative of FoxP3-positive cells. Red staining is indicative of PD-L1-positive cells. Blue staining is indicative of all cells. Scale bar, 200μm.

Varying degrees of T cell infiltration were observed in the collected medulloblastoma tissue samples. The representative immunofluorescence images are shown in **Figure 1**. The results showed that the median proportion of the total T cells was 2.03% (IQR: 0.77%, 5.03%), CD8 T cells was 0.93% (IQR: 0.30%, 2.90%), CD4 T cells was 0.69% (IQR: 0.29%, 1.37%), and CD4^+^FoxP3^+^ cells was 0.04% (IQR: 0.02%, 0.12%). In addition, it was observed that the majority of these patients exhibited the expression of PD-1/PD-L1, which was relatively low. The expression of PD-1 and PD-L1 was 0.15% (IQR: 0%, 0.75%) and 0.08% (IQR: 0.01%, 0.49%), respectively. The proportion of CD3^+^CD8^+^PD-1^+^ cells was 0.11 (IQR: 0%, 0.50%). Among the analyzed cases, 14 (19%) exhibited a proportion of PD-1^+^ cells greater than 1%, while 7 (10%) exhibited a proportion of PD-L1^+^ cells greater than 1%. Additionally, PD-1 expression was undetectable in 19 cases (26%), and PD-L1 expression was not observed in 16 cases (22%).

**Figure 2:**
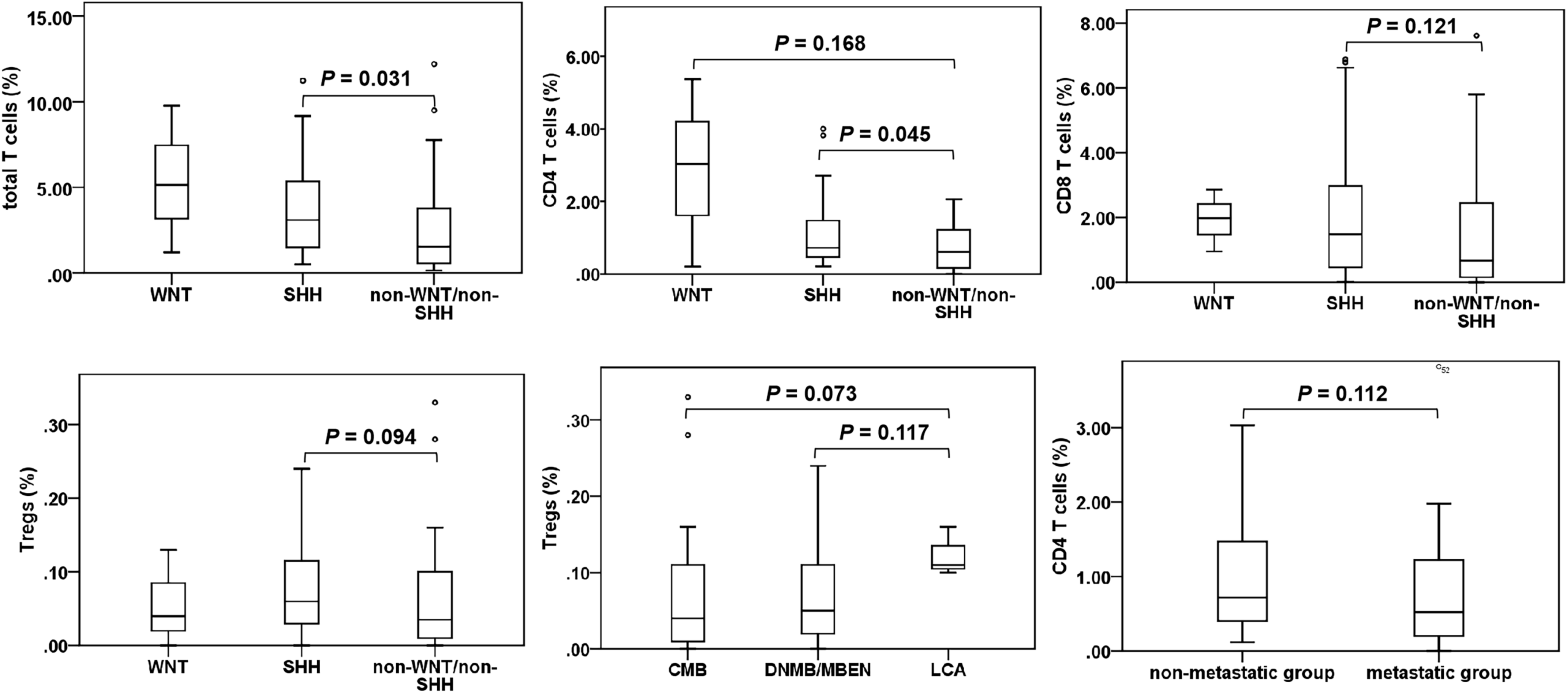
T cell subgroups in medulloblastoma patients. Only significant results and tends are presented. °, outlier.

We analyzed and compared the proportions of various T cell subgroups and PD-1/PD-L1 expression in patients exhibiting diverse clinical characteristics, as detailed in **Table 1** and **Figure 2**. It was showed that the total T cells (*P* = 0.031) and CD4 T cells (*P* = 0.045) were significantly elevated in the SHH subgroup compared to those in the non-WNT/non-SHH subgroup. Additionally, CD8 T cells and Tregs in the SHH subgroup showed an increasing trend. CD4 T cells in the WNT subgroup demonstrated an increasing trend compared to the non-WNT/non-SHH subgroup. Tregs in the LCA group also exhibited an increasing trend. Furthermore, CD4 T cells in the non-metastatic group exhibited an upward trend. There was no significant association between other clinical characteristics and T cells, as well as PD-1/PD-L1 expression (*P* > 0.05).

### Association between T cells and survival in medulloblastoma

**Figure 3:**
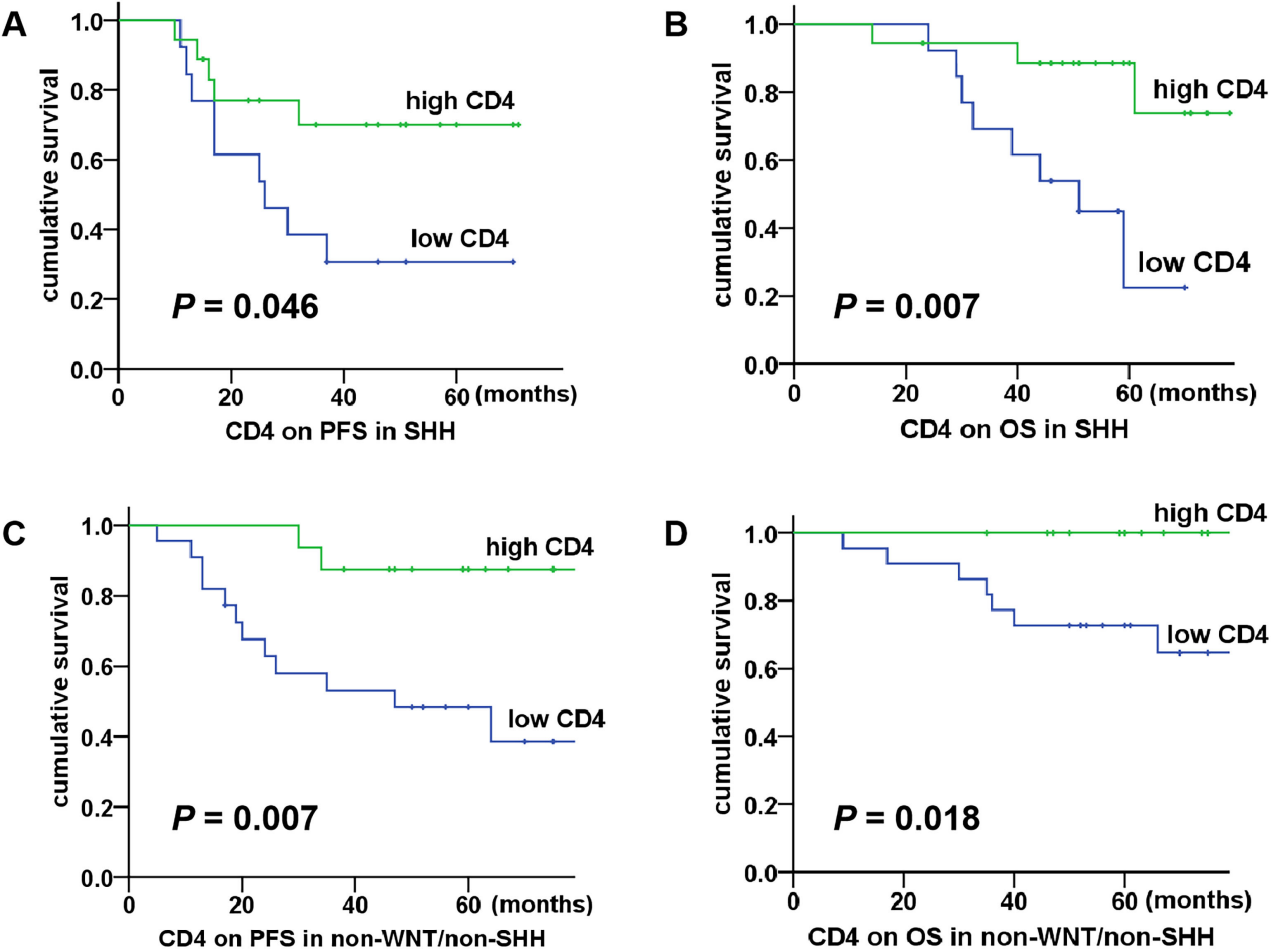
Association between CD4 T cells and survival in SHH and non-WNT/non-SHH medulloblastoma. **(A, B)** The high CD4 T cell group had better 5-year PFS and OS in the SHH subgroup (*P* = 0.046, *P* = 0.007); **(C, D)** The high CD4 T cell group had better 5-year PFS and OS in the non-WNT/non-SHH subgroup (*P* = 0.007, *P* = 0.018).

The correlation between T cells, PD-1/PD-L1 expression and clinical characteristics in relation to patient prognosis was further analyzed, as shown in **Table 2**. According to the median proportions of T cells and PD-1/PD-L1 expression (CD3: 2.03%, CD8: 0.93%, CD4: 0.69%, Tregs: 0.04%, PD-1: 0.15%, PD-L1: 0.08%), the children were divided into two groups: the high proportion group and the low proportion group. The results of the univariate analysis showed that patients with increased CD4 T cells had better 5-year PFS (*P* = 0.000) and OS (*P* = 0.001). Additionally, non-metastatic group showed better 5-year PFS (*P* = 0.031) and OS (*P* = 0.015). Consequently, CD4 T cells and metastasis were identified as significant prognostic factors influencing both the 5-year PFS and OS. There was no statistically significant correlation between other T cells and clinical features in relation to the 5-year PFS or OS (P > 0.05). The results of the multivariate analysis demonstrated that CD4 T cells were an independent prognostic factor affecting both the 5-year PFS (*P* = 0.004, HR = 0.230, 95% CI = 0.085-0.662) and OS (*P* = 0.017, HR = 0.180, 95% CI = 0.044-0.739), which were identified as a protective factor for the prognosis of children. Subsequently, we conducted a further analysis of the correlation between CD4 T cells and the prognosis of medulloblastoma molecular subgroups. The results demonstrated that within both the SHH subgroup and the non-WNT/non-SHH subgroup, patients in the high CD4 T cell group exhibited significantly improved 5-year PFS (*P* = 0.046 for SHH; *P* = 0.007 for non-WNT/non-SHH) and OS (*P* = 0.007 for SHH; *P* = 0.018 for non-WNT/non-SHH), as shown in **Figure 3**. The WNT subgroup was not subjected to analysis due to the limited number of cases.

**Table 2:** Univariate and multivariate analyses of clinical variables associated with PFS and OS.

Factors	PFS				OS			
	Univariate	Multivariate			Univariate	Multivariate		
	*P*	*P*	HR	95% CI	*P*	*P*	HR	95% CI
CD3	0.259	0.319	0.443	0.090 - 2.195	0.164	0.320	0.381	0.057 - 2.553
CD8	0.813	0.265	2.174	0.554 - 8.527	0.953	0.191	2.456	0.639 - 9.440
CD4	0.000	0.004	0.230	0.085 - 0.662	0.001	0.017	0.180	0.044 - 0.739
Tregs	0.568	0.556	1.286	0.557 - 2.969	0.371	0.381	1.614	0.553 - 4.708
PD-1	0.950	0.243	2.047	0.615 - 6.810	0.734	0.544	1.546	0.378 - 6.331
PD-L1	0.508	0.381	0.683	0.292 - 1.601	0.361	0.548	1.404	0.464 - 4.244
Age	0.316	0.651	1.243	0.485 - 3.184	0.107	0.269	2.353	0.515 - 10.744
Metastasis	0.031	0.132	1.804	0.838 - 3.884	0.015	0.114	2.175	0.829 - 5.702

HR, hazard ratio; CI, confidence interval.

### Association between T cells and TAMs in medulloblastoma

We have previously investigated the role of TAMs in this patient population, which can be categorized into different phenotypes (M_total_, M1, M2 and M_mix_). M_total_ represents the total macrophages, and M_mix_ represents the mixed phenotype macrophages. We conducted Spearman correlation analysis to evaluate the relationships between T cells and TAMs, as well as PD-1/PD-L1. The detailed results are presented in **Table 3**. It was observed that CD4 T cells exhibited a positive correlation with M_total_ (*P* < 0.05, r = 0.249) and M_mix_ (*P* < 0.01, r = 0.325). Additionally, CD3^+^CD8^+^PD-1^+^ cells showed a positive correlation with M_mix_ (*P* < 0.05, r = 0.258). However, these correlations were relatively weak. There was no significant correlation between the other cell types (*P* > 0.05). The results suggested a potential association between CD 4 T cells and macrophage infiltration, as well as between CD3^+^CD8^+^PD-1^+^ cells and M_mix_.

**Table 3:** Spearman correlation analysis between T cells and TAMs.

Facors	CD3	CD8	CD4	Tregs	PD-1	PD-L1	CD3^+^CD8^+^PD-1^+^
Mtotal	0.095	0.040	0.249*	-0.063	0.135	0.053	0.146
M1	0.091	0.082	0.080	-0.058	0.117	0.122	0.120
M2	0.035	-0.022	0.109	-0.029	0.097	-0.144	0.119
M_mix_	0.193	0.169	0.325**	0.108	0.228	0.018	0.258*

The values presented in the table represent Spearman correlation coefficients (r). **P < 0.01,*P < 0.05.

## Discussion

At present, the relationship between T cell infiltration and the prognosis of pediatric medulloblastoma remains unclear. Our study demonstrated that the total T cells and CD4 T cells increased in the SHH subgroup, and an increase in CD4 T cells predicted a better 5-year PFS and OS in medulloblastoma. Additionally, our findings suggested a potential interaction between CD4 T cells and macrophages.

Research has demonstrated that T cells are recruited to the medulloblastoma TME only after the tumor cells have interacted with tumor vascular endothelium (17). Among these interactions, macrophage migration inhibitory factor (MIF) plays a crucial role as a chemokine, promoting the secretion of potent T lymphocyte attractant by tumor vascular endothelial cells. In medulloblastoma, T cell infiltration is predominantly observed within intratumor and perivascular regions, and the SHH subgroup exhibits a comparatively higher density of T cells (2, 9). Our research revealed a heterogeneous distribution of T cells within medulloblastoma tissues, with a notable predominance of CD3^+^CD8^+^ T cells. Additionally, the SHH subgroup exhibited a significantly higher infiltration of T cells. These findings are consistent with previously published literature above. Pham CD et al. (9) reported a higher abundance of CD4 and CD8 T cells in the SHH subgroup. Bockmayr M et al. (2) observed that CD8 T cells were more enriched in Group 3, whereas Chai X et al. (18) noted that the CD8 T cells exhibited a higher degree of infiltration in the WNT subtype compared to other subtypes. However, our study showed an increase in CD4 T cells specifically within the SHH subgroup, while no significant differences were observed in other types of T cells across the molecular subtypes. Consistent with previous reports (9, 12), our findings indicated that there were no variations in T cell infiltration with respect to age, pathological types, metastatic status, or other clinical characteristics. Interestingly, our analysis revealed several differential trends. CD8 T cells in the SHH subgroup exhibited an increasing trend, which is consistent with Pham CD’s study. CD4 T cells in the WNT subgroup demonstrated an increasing trend, and Tregs in the LCA group exhibited an increasing trend. However, further investigation in WNT subgroup and LCA group is warranted due to the limited number of cases. Additionally, an increasing trend in Tregs within the SHH group also deserves further attention. An upward trend in CD4 T cells within the non-metastatic group suggests a potential anti-tumor effect mediated by CD4 T cells.

In this study, we identified a significant association between CD4 T cells and favorable prognosis in medulloblastoma patients. Furthermore, we confirmed a same association in both SHH and non-WNT/non-SHH subtypes, which is consistent with the findings in the overall population. Due to the limited number of cases in the WNT group, a prognostic analysis was not performed. Nevertheless, given the increasing trend of CD4 T cells observed in WNT cases, we speculate that the prognosis may also be consistent. However, further research is warranted to incorporate more cases. Nevertheless, our analyses did not uncover any statistically significant associations between other T cell types and the prognosis of medulloblastoma patients. Vermeulen JF et al. (12) have demonstrated that there is no significant correlation between various types of T cells and the overall survival in medulloblastoma. Murata D et al. (13) have shown that a reduction in CD8 T cells predicts a poor prognosis for patients with medulloblastoma, but the number of cases was relatively limited, comprising only 16 cases. Recently, a study has revealed that patients with more infiltration of CD8 T cells have better prognosis (18). The reason for the different results may be related to the heterogeneity of the disease, with different proportions in terms of pathological types and molecular subtypes. Furthermore, recent advancements in medulloblastoma have introduced more detailed molecular categorizations (19), which may explain the observed discrepancies. One of the limitations of this study is the current sample size, which may not fully represent the population. Future research should strive to expand the sample size and perform more detailed molecular subgrouping to enhance the robustness and generalizability of the findings.

Currently, the underlying anti-tumor mechanism of CD4 T cells in medulloblastoma remains undefined. In Pham CD’s study (9), a significant increase in CD4 T cells was observed in the medulloblastoma mouse models which were effective to anti-PD-1 treatment. They indicated that CD4 T cells may drive the antitumor response as antigen-specific T cells. Research has demonstrated that CD4 T cells can kill autologous tumors in an MHC class II-dependent fashion (20). However, CD4 cells also have different subtypes and functions (21), which our study did not further distinguish. This is a limitation of our study and warrants further investigation. Our findings offer valuable insights for therapeutic strategies, such as finding ways to increase the influx of beneficial CD4 T cells into the TME of medulloblastoma.

The PD-1/PD-L1-mediated immune checkpoint in the TME is an important component of the tumor immune escape mechanism. T cells can recognize tumor cells and exert cytotoxic effects on them. However, when tumor cells recognize PD-1 on T cells, they upregulate PD-L1 expression, and the binding of PD-1 and PD-L1 leads to the apoptosis of T cells (22). It is reported that the number of PD1^+^ T cells is limited in medulloblastoma, and no expression of PD-L1 was detected (12). Murata et al. observed that in 16 medulloblastoma samples, 9 cases showed high expression of PD-L1 (13). In this study, we observed the expression of PD-1/PD-L1 in the majority of patients; however, the overall expression rate was relatively low. Specifically, approximately 20% of these cases exhibited PD-1^+^ cells exceeding 1%, while around 10% exhibited PD-L1^+^ cells greater than 1%. Furthermore, a subset of patients demonstrated undetectable expression of PD-1 (27%) or PD-L1 (23%). Research has indicated that elevated expression of PD-L1 is associated with adverse prognosis in medulloblastoma patients (13). PD-1/PD-L1 can also serve as a predictive marker for clinical response to topical immunotherapy (23). However, our study did not identify any significant correlation between PD-1/PD-L1 expression and the prognosis in pediatric patients.

We analyzed the correlation between T cells and TAMs, and noted that CD4 T cells were positively correlated with the total macrophages and M1/M2 mixed phenotype cells. In addition, CD3^+^CD8^+^PD-1^+^ cells were positively correlated with M1/M2 mixed phenotype cells. However, the correlations were relatively weak. The specific mechanism requires more in-depth investigation. Studies have demonstrated that TAMs are associated with exhausted CD8^+^ cells (24, 25). The depletion of TAMs reduces the exhaustion programs in tumor-infiltrating CD8 T cells and reinvigorates their effector potential. Reciprocally, exhausted T cells secrete factors that actively recruit monocytes to the TME and influence their differentiation. Inhibition of the CCL2-CCR2 axis can effectively reduce tumor incidence by impeding the recruitment of TAMs, consequently augmenting the anti-tumor effect of CD8 T cells (26). The literature has reported the important function of the CD4^+^ Th1/TAM axis in re-regulating the immunosuppressive TME (27). The homologous interaction between CD4^+^ Th1 cells and TAMs may shift the M1/M2 ratio within tumors towards M1. In other types of tumors, a correlation has been reported between M1 macrophage infiltration and PD-L1 expression (28). In this study, the observed weak correlation between CD3^+^CD8^+^PD-1^+^ cells and M1/M2 mixed phenotype suggests a potential synergistic interaction; however, the precise mechanisms underlying this relationship require further research.

## Conclusion

The increase in CD4 T cells predicts a better prognosis in medulloblastoma patients, particularly within the SHH and non-WNT/non-SHH subgroups, and they might be taken as a therapeutic target for medulloblastoma. Additionally, there may exist a potential interaction between CD4 T cells and TAMs.

## Data Availability

The original contributions presented in the study are included in the article/supplementary material. Further inquiries can be directed to the corresponding authors.
